# To Whom It May Concern

**Published:** 1888-04

**Authors:** 


### TO WHOM IT MAY CONCERN.

We are in receipt of letters from a number of dentists who enquire about "The Porcelain Dental Art Company," of Detroit, and say that our name has been published as a licensee of the company. We desire to make this public answer to save the necessity for personal communications.

For some years we have been using one of Dr. Land's Gas Furnaces, for baking continuous gum work and for other laboratory purposes, and always with increasing satisfaction. We believe that it will do quite as good work as any of the coke furnaces, and at a great saving of time and trouble. This furnace was bought long anterior to the formation of any company for its control, and entirely without stipulations.

Of "The Porcelain Dental Art Company" we have no knowledge whatever, and have never had dealings with it in any manner. We have no license for the use of patented privileges of any kind, and want none. We have never given authority for the use of our name in connection with any system of licenses, and most decidedly disapprove of all such methods among those who call themselves professional men. If we cannot use our gas furnace without a special license, we shall most certainly lay it aside henceforth and forever.

